# A Clinical-EEG Study of Sleepiness and Psychological Symptoms in Pharmacoresistant Epilepsy Patients Treated with Lacosamide

**DOI:** 10.1155/2013/593149

**Published:** 2013-09-19

**Authors:** Filippo S. Giorgi, Chiara Pizzanelli, Veronica Pelliccia, Elisa Di Coscio, Michelangelo Maestri, Melania Guida, Elena Iacopini, Alfonso Iudice, Enrica Bonanni

**Affiliations:** ^1^Neurology Unit and Epilepsy Center, Department of Neuroscience, A.O.U.P and Department of Clinical and Experimental Medicine of the University of Pisa, Via Roma 67, 56126 Pisa, Italy; ^2^Sleep & Epilepsy Center, Neurocenter of the Civic Hospital (EOC) of Lugano, Via Tesserete 46, 6900 Lugano, Switzerland

## Abstract

Our aim was to evaluate the EEG and clinical modifications induced by the new antiepileptic drug lacosamide (LCM) in patients with epilepsy. We evaluated 10 patients affected by focal pharmacoresistant epilepsy in which LCM (mean 250 mg/day) was added to the preexisting antiepileptic therapy, which was left unmodified. Morning waking EEG recording was performed before (*t*0) and at 6 months (*t*1) after starting LCM. At *t*0 and *t*1, patients were also administered questionnaires evaluating mood, anxiety, sleep, sleepiness, and fatigue (Beck Depression Inventory; State-Trait Anxiety Inventory Y1 and Y2; Pittsburgh Sleep Quality Index; Epworth Sleepiness Scale; Fatigue Severity Scale). We performed a quantitative analysis of EEG interictal abnormalities and background EEG power spectrum analysis. LCM as an add-on did not significantly affect anxiety, depression, sleepiness, sleep quality, and fatigue scales. Similarly, adding LCM to preexisting therapy did not modify significantly patient EEGs in terms of absolute power, relative power, mean frequency, and interictal abnormalities occurrence. In conclusion, in this small cohort of patients, we confirmed that LCM as an add-on does not affect subjective parameters which play a role, among others, in therapy tolerability, and our clinical impression was further supported by evaluation of EEG spectral analysis.

## 1. Introduction

Epilepsy is one of the most common neurological disorders, affecting up to two percent of the population worldwide. Many patients show recurrent seizures despite treatment with appropriate antiepileptic drugs (AEDs') [1, 2], and many experience AEDs side effects. In the last decades, new AEDs have been developed with the aim of balancing, as far as possible, significant efficacy with good tolerability.

Among them, Lacosamide (LCM) has been recently authorized in Italy and worldwide as a new add-on AED for the treatment of pharmacoresistant focal epilepsy.

Side effects of classical AEDs often involve cognitive functions, mood, and behavior to varying degrees, and this is the case also for newer AEDs (see, for instance, [3–5]). Unfortunately, a clear evaluation of these types of side effects in the single patient is often difficult because of the subjectivity of such complaints. This assessment is even harder in patients undergoing AED polytherapy.

It has been proposed by several authors the usefulness of a quantitative analysis on EEG in patients undergoing treatment with drugs acting on the CNS (for a review, see for instance, [6]), in this setting, abnormalities of EEG power spectrum have been interpreted as an objective measure of cognitive slowing/impairment (see, for instance, [7–9]). Furthermore, in the last decades, questionnaires specifically evaluating mood, anxiety trait, sleepiness, fatigue, and sleep quality have been developed.

The aims of the present study were (i) to analyze the effects of LCM on EEG in terms of EEG background spectra and interictal activity and (ii) to further evaluate LCM effects by using subjective questionnaires addressing depression (Beck Depression Inventory-BDI), anxiety (State-Trait Anxiety Inventory-STAI), sleep quality (Pittsburgh Sleep Quality Index-PSQI), sleepiness (Epworth Sleepiness Scale-ESS,) and fatigue (Fatigue Severity Scale-FSS).

## 2. Materials and Methods

### 2.1. Patients and Study Design

Ten patients affected by focal epilepsy (6 males and 4 females, mean age 48.2 ± 14.8 years) were included in this study. The mean age at epilepsy onset was 13.5 ± 7.9 years. Five patients were affected by focal symptomatic epilepsy, and five were affected by probably focal symptomatic epilepsy. In Table 1, we reported a detailed description of etiologies, electroclinical features, and concomitant AEDs, as well as comorbidities. 

The design of this study is a prospective open-label pragmatic one. We selected ten consecutive adult outpatients from our tertiary University Epilepsy Center who were fulfilling the following criteria: (a) being affected by partial focal epilepsy, not caused by a progressive etiology; (b) having experienced in the previous three months at least 12 seizures (not less than 2 for each single month); (c) having been treated with more than one appropriate AED, at adequate dose regimen; and (d) being screened for any kind of AV Block by at least a routine EKG. 

Recruited Patients were submitted to video-EEG recording and clinical evaluation on the day before (*t*0) and at 6 months (*t*1) after beginning LCM. 

LCM was administered to all of the enrolled patients at a starting dose of 50 mg/day, followed by biweekly 50 mg/day dose increase, up to each patient's maintenance dose on the basis of clinical response and tolerability (mean final daily dosage of 250 ± 81.6 mg/die). The remaining AED therapy was left unmodified throughout the study: in 9 patients, this included AEDs acting on voltage-gated Na+ channels (Table 1). Neurological examination and blood tests, including AED plasma levels, were monitored at *t*0 and *t*1; during these same visits, patients were administered the subjective questionnaires that were selected also based on previous studies in epilepsy patients [10–12] and are detailed below. 

Starting at six months before *t*0, patients were asked to collect a detailed seizure diary, which were collected by the examiner at *t1*. 

As shown in Table 2, there was a seizure reduction of 33.3% at *t*1 versus *t*0. In particular, 7/10 patients showed a seizure reduction at *t*1; in one, there was a slight seizure increase; four patients showed a seizure reduction ≥50%, and one of them was seizure-free at *t*1. When comparing raw seizure number at *t*1 versus *t*0, *P* was 0.068.

### 2.2. EEG Procedures

Each patient was admitted at our Sleep-Epilepsy Center for video-EEG monitoring session at *t*0 and *t*1 (see above).

Participants were instructed to follow their usual daily routine, meals, and caffeine consumption and to refrain from alcohol intake for 24 h before starting the recording. The EEG recordings were performed through a 32-channel cable video-telemetry system. Nineteen collodion-applied scalp-electrodes were placed according to the 10–20 system; chin electromyogram, electrocardiogram, and electrooculogram signals were recorded via additional skin surface electrodes. Electrode impedance was maintained below 5 kΩ. Filters were set at 0.1 and 30 Hz, and signal was notch filtered. Two additional electrodes were placed at mastoid level; for spectral analysis, only O1-mastoidal and O2-mastoidal traces were considered. All the EEG recordings were carried out with the same type of digital EEG equipment (BElite, EBNeuro, Florence), and data were acquired with a 258 bit sampling rate and stored on the PC hard disk for offline evaluation. 

The EEG was recorded in a silent room of the University Sleep Center, during constant monitoring by an EEG technician. 

The recording periods included (a) a night recording (polysomnography-PSG) from 9 p.m. to 7 a.m. of the following day (not shown) and (b) a routine video-EEG wake recording from 8 to 9.30 a.m. after the end of PSG.

On the morning 1 .5 h video EEG recording, we performed an analysis of interictal epileptiform abnormalities (IIA) and power spectrum analysis of background activity. 

In detail, we performed the following analysis of EEG data.

#### 2.2.1. Interictal Abnormalities (IIA) Analysis

 IIA occurrence was analyzed visually by two independent observers which were blinded, for each patient, as to whether they were scoring a *t*0 or *t*1 EEG tracing. The total number of IIA occurring during the 8−9.30 A.M. wake-EEG was recorded and converted to *n*/10′. 

#### 2.2.2. Power Spectrum Analysis

 Epoch selection for qEEG analysis was performed offline on waking EEG recording obtained from 8 to 9.30 A.M. We selected randomly, and blindly to patient number and treatment, EEG periods lacking ictal and/or interictal abnormalities, movements artifacts, eye blinking, muscle activity or drowsiness signs. On these EEG parts, we used the fast Fourier transform (FFT), considering 2 minutes of EEG signal, automatically segmented by software into 2.56 s epochs. Analysis was performed for each frequency band: delta [1–4 Hz]; theta [4–8 Hz]; alpha [8–12 Hz], and beta [12–30 Hz]. 

Measures derived from FFT included (i) absolute power; (ii) percent relative power, and (iii) mean frequency. 

We chose to analyze mainly the frequency in occipital derivation according to widely accepted criteria [6]. Moreover, the analysis of occipital recording allows the best identification of alpha activity, and recordings are devoid of artifacts observed in more anterior leads. To minimize statistical problems associated with multiple variables, results from the O1 and O2 leads were averaged for analysis. 

### 2.3. Subjective Questionnaire

In order to evaluate the wake-sleep symptoms and psychological well-being of the patients included in this study, five scales were administered before and after 6 months of LCM therapy.

#### 2.3.1. Beck Depression Index

 BDI is a small questionnaire examining 21 symptom areas with a total score ranging from 0 to 63 proportionally to depression severity [13]. 

#### 2.3.2. The State-Trait Anxiety Inventory

STAI is a brief self-administered questionnaire for the assessment of state and trait anxiety in adults and is composed of a State anxiety scale (STAI Y-1) and a Trait anxiety scale (STAI Y-2), consisting of 20 statements each [14].

#### 2.3.3. Epworth Sleepiness Scale

ESS is the subjective scale that is generally considered as the gold standard for the evaluation of daytime sleepiness [15]. It evaluates individual degree of drowsiness in eight common daily conditions, has been validated in Italian [16], and is widely used in epilepsy [17]. It is generally accepted a cutoff of 10 as normal value [18]. 

#### 2.3.4. The Pittsburgh Sleep Quality Index

 PSQI is an instrument used to measure the quality and patterns of sleep in adults assessing seven domains self-rated by the subject [19] and already used also in epilepsy patients [20]. A global score of 5 or more reveals a poor quality of sleep and is considered as the cutoff from normal to pathological values.

#### 2.3.5. The 9-Item Fatigue Severity Scale

(FSS) is one of the most commonly used self-report questionnaires to measure fatigue [21] with value ranging from 1 (strong disagreement with the statement) to 7 (strong agreement). A cut-off of 4 is generally considered [22].

### 2.4. Statistical Analysis

For IIAs, absolute power spectrum (for each frequency band), relative power spectrum (for each frequency band), mean alpha frequency, and seizure frequency, a Student's *t*-test analysis for paired data was applied to compare *t*1 and *t*0 data.

For scales (STAI, BDI, ESS, FSS, and PSQI), the score comparisons between *t*1 and *t*0 were performed by the Wilcoxon signed-rank test.

For all of the analyses, the null hypothesis was rejected when *P* < 0.05.

## 3. Results

### 3.1. Adverse Effects of LCM

LCM was not discontinued in any of the patients. Five patients complained mild drowsiness, and one patient experienced sleepiness, but these effects were transient and improved right after slowing the titration schedule. Blood levels of concomitant AEDs were not significantly affected by LCM administration (not shown). 

### 3.2. Effects of LCM on EEG IIAs (Table 2)

As shown in Table 2, in all but two patients we observed either a decrease or a lack of effect of LCM on IIAs. Patient 1 already at baseline showed a significantly higher IIA number (22.2) than the remaining ones (1.72 ± 0.65), and at *t*1, there was 14% increase in its occurrence. With the exception of these two patients, in all of the remaining ones there was no effect or a slight decrease in IIAs occurrence. The mean IIAs % change at *t*1 was −19.3% versus baseline.

### 3.3. Effects of LCM on qEEG (Table 3 and Figure 1)

**Figure 1:**
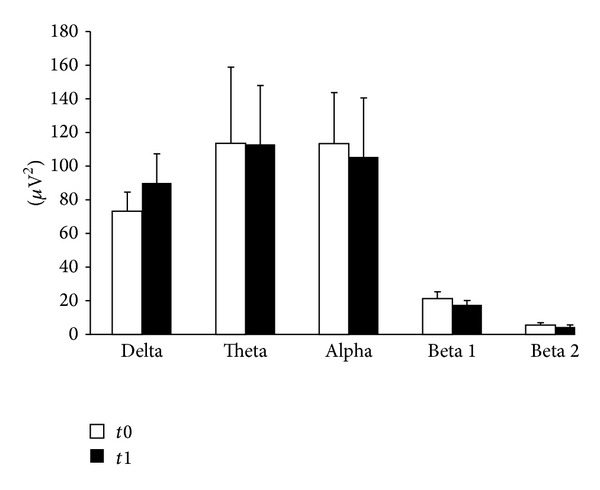
Power spectrum analysis of EEG. Patients were assessed at t0 and after 6 months (*t*1). The graph shows absolute power (*μ*V^2^) calculated on O2-Ref EEG traces. qEEG analysis was performed offline on waking EEG recording obtained from 8 to 9.30 A.M., randomly selecting EEG periods lacking ictal and/or interictal abnormalities, movements artifacts, eye blinking, muscle activity or drowsiness signs. On these EEG parts, we used the fast Fourier transform (FFT), considering 2 minutes of EEG signal, automatically segmented by software into 2.56 s epochs. Analysis was performed for each frequency band: delta [1–4 Hz]; theta [4–8 Hz]; alpha [8–12 Hz], and beta [12–30 Hz]. None of these bands were significantly affected by LCM treatment.

Concerning qEEG, LCM did not significantly affect the absolute power density for any of the frequency intervals evaluated (Figure 1, Table 3), apart from a slight, nonsignificant increase in delta frequency representation. Similarly, alpha mean frequency was not affected by LCM administration, as well as the mean frequency of the remaining bands (Table 3).

PSG data concerning the night before EEG recording were analyzed in detail and are part of a separate multicenter study (in preparation); in any case, all PSG recordings showed a total sleep time longer than 6 hours, which is considered necessary for a proper evaluation of sleepiness in international guidelines [23], and no statistical differences were found between *t*0 and *t*1 for the variables of sleep continuity (i.e. sleep efficiency, total sleep time).

### 3.4. Psychological Effects (Tables 4 and 5)

We did not observe any significant changes in BDI scores. Nevertheless, in four patients with intermediate BDI scores, we observed an improvement at *t*1 (patients 1, 2, 4 and 7). In patient #6 presenting a high BDI score at *t*0 (22), we did not observe any changes at *t*1.

Similarly, in the STAI scales the scores remained stable throughout the observation period.

At baseline, excessive daytime sleepiness (ESS score ≥10) was reported by two patients. Six months after LCM therapy, two other patients had pathological scores at ESS. However, no patients reported severe daytime sleepiness, that is, ESS > 14, and when measuring the group mean values, no statistical differences were observed. Also, FSS showed no differences between *t*1 and *t*0, even if it was higher than normal ranges in both conditions. Concerning PSQI, the percentage of “good sleepers” (i.e., with a score <5) was 50% at *t*0 and 80% at *t*1.  Table 5 shows mean values for each PSQI subitem at *t*0 and *t*1.

## 4. Discussion

In this small cohort of pharmacoresistant focal epilepsy patients, we investigated the effects of LCM in terms of EEG and psychological effects. We showed that LCM does not affect significantly EEG background in terms of power spectra, nor does it worsen depressive or anxiety traits, as well as subjective indices of sleepiness, fatigue, and sleep quality in this type of patients. Furthermore, LCM did not affect IIAs occurrence significantly, despite its efficacy on seizures. 

We chose a prolonged observation period (6 months from *t*0 to *t*1) in order to allow (a) a prolonged slow titration of the LCM; and (b) a complete stabilization of the effects of the drug on both EEG and clinical conditions.

The effect of LCM was much lower on IIAs than toward seizures and not remarkable. This is not surprising, since previous studies failed to show a parallelism between seizure and IIAs frequency concerning other AEDs, such as carbamazepine [24] and gabapentin [25] in focal epilepsy. Incidentally, an elegant experimental study performed in amygdala kindled cats confirmed the lack of an effect of carbamazepine on spike occurrence, despite a significant effect on seizures [26]. Conversely, topiramate [27] and lamotrigine [28] have been shown to reduce IIAs incidence and spreading, in parallel with seizure occurrence. The lack of a correlation between IIAs reduction and seizure frequency we observed in our study might be due to the pharmacodynamic effects of LCM itself. However, further experimental studies would be needed to address this hypothesis.

When deciding to add a new AED in pharmacoresistant epilepsy patients, the main concern of the prescribing physician is to get the maximum efficacy with the lowest incidence of side effects. Among the main complaints of pharmacoresistant epilepsy patients in terms of AED tolerability, there are drowsiness, confusion, and dizziness, as well as mood changes and anxiety.

The low incidence of CNS side effects after LCM we found in our study, as well as the complete lack of dropout during follow up, might be due to the design of our study indeed; in fact, the titration of LCM was shaped on patients' tolerability and efficacy, and the long follow-up period (6 months) allows a full stabilization of the appropriate drug regimen.

Several authors have proposed that EEG background correlates with the degree of alertness and of cognitive performances (for a detailed review, see [29]); even though such a link is indirect and difficult to quantify, many investigators agree on a solid correlation of slowing of alpha mean frequency with cognitive impairment (see, for instance, the reviews by [30, 31]). We could not show any significant reduction of alpha activity at *t*1 versus *t*0 nor an increase in the representation of slower frequencies (i.e. delta and theta ones). This is in agreement with the lack of subjective significant complaints reported by our group of patients, both in terms of drowsiness, and in terms of increased cognitive impairment, as compared with baseline. Potentially, the EEG derivations we used (occipital ones) for spectral evaluation are mainly suitable for alpha band analysis, as compared with lower frequency bands, while for slower bands the analysis of additional derivations might be preferable, and this might be a limitation of our study. However, nevertheless we chose to focus on occipital derivations since these are the only ones used also in many similar studies in which significant EEG modifications were observed during AEDs treatment (e.g., [7–9]), while data assessing also other derivations in these types of studies are more sparse and difficult to compare with each other.

Previous studies assessing power spectral analysis in epileptic patients were performed in focal epilepsy populations, either drug free [31] or during AED monotherapy [7, 9, 32–34] or polytherapy [35]. Further, AEDs effects on EEG background have been evaluated also in healthy volunteers [8, 36].

In our study, we did not find, for anyone of the frequency band tested, a difference between *t*0 and *t*1, with this being in line with the effect of other AEDs, such as phenobarbital, lamotrigine, and valproic acid [28, 33, 35]. Conversely, previous studies in patients treated with the sodium-channel blocking AEDs CBZ and OXC showed a decrease of alpha mean frequency [7, 24, 33]. Thus, our findings suggest that the enhancing effects of LCM on voltage-gated sodium channels slow inactivation affects neocortical rhythm in a different manner as compared with the effect of fast-inactivation enhancement. The lack of any significant effect we observed on qEEG might have been due to the fact that already at baseline EEG background was significantly affected both by the underlying disease and by the concomitant AEDs. However, indeed our aim was not to compare our data with those of a control population (since our subjects were not healthy volunteers taking LCM) but to show, if any, the existence of a worsening potential effect of LCM on EEG background in the particular population of patients who are affected by pharmacoresistant epilepsy. Furthermore, we found mean variability in the different frequency bands similar to those observed by other authors in AED monotherapy (see for instance [7, 33, 34]).

AEDs bear, to varying degrees, psychological effects including effects on mood and anxiety [3, 37, 38]; furthermore, it has been shown that the incidence of such adverse effects increases in parallel with the number of ongoing AEDs [3]. In this study, patients were administered with BDI to address depressive features, which is a well-validated scale that has been used extensively in such populations before [10, 39]; this scale was not significantly affected by adding LCM. Anxiety is another one of the commonest complaints in patients undergoing antiepileptic therapy (see [3, 5]); we showed that STAI questionnaires, which explore anxiety trait and state and have been validated in several populations affected by chronic neurological illnesses [39–41], are not modified by LCM add-on. However, it should be noted that both depressive and anxiety features at *t*0 were slightly elevated in our patients as compared to control populations from our lab historical data (not shown). This might affect the finding of no effect of LCM add-on on these measures. However, as said, the aim of this study was to assess, indeed, the additional effect of LCM on a category of patients already bearing a burden of potential side effects of different drugs and of the disease itself as well.

Concerning sleep-wake cycle, our study shows that subjective standardized scales did not highlight significant changes when LCM was added to previous therapies. As concerns sleepiness, in the registration studies, LCM showed a risk of sleepiness as a side effect (3.1% when considering differences towards placebo), which is lower than other new AEDs (see as a review, [42]). An exhaustive discussion about subjective evaluation of sleep, sleepiness, and fatigue in pharmacoresistant epilepsy patients is complex and far beyond the aim of this study; it is worth noting that the ESS and PSQI scores in our patients are within normal range, while FSS showed higher levels of fatigue than usually reported in general population, but without statistically significant changes during LCM therapy. 

A discrepancy between objective and subjective evaluation of sleep and sleepiness in epilepsy has been suggested, and we could hypothesize that single patients could underestimate the degree of these disturbances, since these could be chronic symptoms, and subjects could be more focused on seizure frequency and on daytime fatigue. Moreover, the subjective differentiation between sleepiness and fatigue is complex and not completely understood ([43]). 

Thus, a study using objective standardized methods (i.e. polysomnography and multiple sleep latency test) to evaluate sleep and sleepiness would be necessary to further understand the impact of LCM on these aspects. 

## 5. Conclusions

In this study, we observed that our clinical impression of tolerability of LCM as an add-on was further significantly supported by objective EEG measures and by semiquantitative analysis of effects on sleepiness, mood, and anxiety, even though therapy tolerability as a whole is due also to many aspects not specifically evaluated in this paper. 

 We are aware that this study was not randomized in design and the patients were under previous AEDs. However, since we compared the chronic effects of LCM versus each patient's own baseline and throughout an observation period of 6 months, this makes our findings interesting, since they reflect closely a typical clinical setting of patients taking LCM.

## Figures and Tables

**Table 1:** Demography.

Patients	Age	Age at onset (yr)	Epileptic syndrome	Seizure type	AEDs before-LCM
No. 1	45	11	Symptomatic temporal lobe epilepsy (posttraumatic)	Focal limbic seizures, SG	OXC 900 mg/die; LEV 3 g/die; VPA 1,5 g/die; LTG 200 mg/die; TPM 600 mg/die; VNS
No. 2	34	13	Symptomatic temporal lobe epilepsy (postradiotherapy calcification)	Focal limbic seizures, SG	OXC 1200 mg/die; LTG 300 mg/die; LEV 3 g/die; VNS
No. 3	34	18	Probably symptomatic temporal lobe epilepsy	Focal limbic seizures, rarely SG	LEV 3,5 g/die; ZNS 200 mg/die; TPM 600 mg/die; CBZ 1600 mg/die
No. 4	76	16	Probably symptomatic frontal lobe epilepsy	Focal seizures	CBZ CR 1200 mg/die; PB 50 mg/die; LTG 200 mg/die; ZNS 450 mg/die
No. 5	43	13	Probably symptomatic temporal lobe epilepsy	Focal limbic seizures	OXC 1800 mg/die; LEV 3 g/die
No. 6	59	6	Probably symptomatic temporal lobe epilepsy	Focal limbic seizures	ZNS 100 mg/die; LEV 1 g × 3/die; OXC 600 mg × 3/die
No. 7	55	10	Symptomatic temporal lobe epilepsy (left HS)	Focal limbic seizures	LEV 2 g/die; OXC 2100 mg/die; TPM 300 mg/die
No. 8	51	25	Symptomatic temporooccipital epilepsy (right retrotrigonal lesion)	Focal limbic seizures	LEV 1 g × 3/die; CBZ 600 + 400 + 600; ZNS 200 mg/die
No. 9	26	1	Symptomatic frontal lobe epilepsy (calcifications of falx cerebri)	Nocturnal frontal seizures	CBZ 1200 mg/die; TPM 450 mg/die
No. 10	59	27	Probably symptomatic temporal lobe epilepsy	Focal limbic seizures	TPM 350 mg/die

HS: hippocampal sclerosis; SG: secondarily generalized; CBZ: carbamazepine; LEV: levetiracetam; LTG: lamotrigine; OXC: oxcarbazepine; TPM: topiramate; VNS: vagus nerve stimulation; VPA: valproic acid; ZNS: zonisamide.

**Table 2:** Interictal EEG abnormalities and seizures frequency after LCM.

Patient	IIAs/10 min *t*0	IIAs/10 min *t*1	% variation IIAs in (*t*1 − *t*0)	Seizures/month *t*0	Seizures/month *t*1	% variation seizure in frequency (*t*1 − *t*0)
No. 1	22,22	25,77	+14	15,16	7,66	−50
No. 2	2,44	1,55	−37	4,66	0	−100
No. 3	1,44	0,77	−45	4	3,16	−21
No. 4	0,33	0,22	−34	22,16	9,16	−59
No. 5	0,88	1	+12	10,16	7,16	−30
No. 6	0,44	0,44	0	3,83	4,33	+12
No. 7	0,77	0,55	−29	4,33	4,33	0
No. 8	0,66	0,44	−34	9,83	7	−29
No. 9	6,66	6,44	−4	54,16	24,16	−56
No. 10	1,88	1,22	−36	3,66	3,66	0

Pooled	3,77 ± 2,13	3,84 ± 2,50	−19,3	13,19 ± 4,94	7,06 ± 2,07	−33,3

Values in bottom row concerning columns 2, 3, 4, and 6 are expressed as mean ± S.E.M.

IIAS: interictal EEG abnormalities.

**Table 3:** Power spectrum analysis of EEG.

	Delta			Theta			Alpha			Beta 1			Beta 2		
	*t*0	*t*1	*P*	*t*0	*t*1	*P*	*t*0	*t*1	*P*	*t*0	*t*1	*P*	*t*0	*t*1	*P*
Absolute power (*μ*V^2^)															
Median	67,145	85,195	0,42	53,5	79,395	0,99	91,52	70,635	0,86	16,125	15,61	0,47	4,165	2,92	0,47
Mean	73,0263	89,97		113,5075	112,9513		113,3288	105,4775		21,1188	17,4675		5,6913	4,3488	
S.E.M.	11,4714	17,2173		45,4567	34,8912		30,1147	34,9935		4,2976	2,4071		1,4159	1,1695	
Relative power															
Median	25,31	28,68	0,61	20,15	26,995	0,81	31,595	28,37	0,82	7,075	5,96	0,49	1,415	1,07	0,45
Mean	26,0737	29,2212		28,2462	30,2275		33,6137	31,845		7,9887	6,3487		2,4275	1,6562	
S.E.M.	4,2267	4,3531		7,0544	4,6986		5,388	5,1308		2,08	1,0074		0,8032	0,5687	
Mean frequency (Hz)															
Median	1,59	1,645	0,99	6,22	6,365	0,74	9,12	9,07	0,73	14,13	13,955	0,12	19,9	19,93	0,99
Mean	1,61	1,6113		6,2087	6,2763		9,3	9,2113		14,1525	13,9425		20,0525	20,0525	
S.E.M.	0,0582	0,0826		0,1541	0,1204		0,18	0,1813		0,0999	0,0816		0,1304	0,1237	

**Table 4:** Psychological effects of lacosamide.

	Score at *t*0 (mean ± S.D.)	Score at *t*1 (mean ± S.D.)	*P*
PSQI	4,4 ± 1,6	3,7 ± 1,3	0.23
ESS	7,7 ± 1,8	8,3 ± 2,4	0.25
FSS	40,4 ± 12,1	36,7 ± 13,5	0.26
BDI	12,1 ± 5,1	9,9 ± 4,4	0.07
STAI Y1	41,1 ± 7,6	39,1 ± 5,9	0.08
STAI Y2	43,7 ± 10,1	42,2 ± 10,9	0.15

Statistical analysis was performed by means of Wilcoxon signed-rank nonparametric test.

BDI: Beck Depression Inventory; ESS: Epworth Sleepiness Scale; FSS: Fatigue Severity Scale; PSQI: Pittsburgh Sleep Quality Index; STAI Y1: S-anxiety scale of the State-Trait Anxiety Inventory form Y; and STAI Y2: T-anxiety scale of the State-Trait Anxiety Inventory form Y.

**Table 5:** Effects of lacosamide on the different subitems of Pittsburgh sleep quality index.

	Score at *t*0 (mean ± S.D.)	Score at *t*1 (mean ± S.D.)	*P*
C1 subjective sleep quality	0,9 ± 0,6	0,7 ± 0,5	0.5
C2 sleep latency	0,4 ± 0,5	0,3 ± 0,5	0.68
C3 sleep duration	0,8 ± 0,4	0,8 ± 0,4	1
C4 sleep efficiency	0,6 ± 0,5	0,5 ± 0,5	0.9
C5 sleep disturbances	1,2 ± 0,8	0,9 ± 0,7	0.34
C6 use of sleeping medication	0,2 ± 0,4	0,3 ± 0,5	0.59
C7 daytime dysfunction	0,3 ± 0,5	0,2 ± 0,4	0.68

Statistical analysis was performed by means of Wilcoxon signed-rank nonparametric test.
